# Salinomycin treatment reduces metastatic tumor burden by hampering cancer cell migration

**DOI:** 10.1186/1476-4598-13-16

**Published:** 2014-01-27

**Authors:** Florian Kopp, Adam Hermawan, Prajakta Shirish Oak, Annika Herrmann, Ernst Wagner, Andreas Roidl

**Affiliations:** 1 Pharmaceutical Biotechnology, Department of Pharmacy, Ludwig-Maximilians-Universität München, Butenandtstrasse 5-13, Munich 81377, Germany

**Keywords:** Salinomycin, Cancer, Migration, Cell motility, Metastasis

## Abstract

**Background:**

Tumor spreading is the major threat for cancer patients. The recently published anti-cancer drug salinomycin raised hope for an improved treatment by targeting therapy-refractory cancer stem cells. However, an unambiguous role of salinomycin against cancer cell migration and metastasis formation remains elusive.

**Findings:**

We report that salinomycin effectively inhibits cancer cell migration in a variety of cancer types as determined by Boyden chamber assays. Additionally, cells were treated with doxorubicin at a concentration causing a comparable low cytotoxicity, emphasizing the anti-migratory potential of salinomycin. Moreover, single-cell tracking by time-lapse microscopy demonstrated a remarkable effect of salinomycin on breast cancer cell motility. Ultimately, salinomycin treatment significantly reduced the metastatic tumor burden in a syngenic mouse tumor model.

**Conclusions:**

Our findings clearly show that salinomycin can strongly inhibit cancer cell migration independent of the induction of cell death. We furthermore demonstrate for the first time that salinomycin treatment reduces metastasis formation *in vivo*, strengthening its role as promising anti-cancer therapeutic.

## Findings

Distant metastases are the major cause of death in patients suffering from cancer. In spite of this, there is a lack of effective treatments for patients with metastatic disease. The discovery and development of novel drugs which can potently inhibit cancer cell migration and hence prevent metastasis formation are therefore of great interest in order to prolong the survival of patients. Gupta *et al*. have recently found salinomycin to be a selective inhibitor of cancer stem cells (CSC) obtained from immortalized transformed HMLER cells by a stable E-cadherin knockdown. Salinomycin reduced the proportion of CSC more than 100-fold as compared to paclitaxel, a commonly used chemotherapeutic breast cancer drug [1]. Subsequent studies in a variety of different cancer types including breast, blood, lung, pancreas and colon have revealed diverse mechanisms of salinomycin action against CSC resulting in an inhibition of proliferation or an induction of apoptosis and cell death [2]. Very recently, some reports have indicated that salinomycin inhibits cancer cell migration in different cancer types [3-8]. However, when looking at these studies in more detail, some of them raise concerns regarding the salinomycin concentration used for the migration experiments. Moreover, the ultimate effect of salinomycin treatment on metastasis formation *in vivo* had yet to be elucidated.

In this study, we wanted to investigate whether salinomycin is able to inhibit migration in a variety of cancer types. In order to rule out that the inhibition of cell motility is due to unspecific cytotoxic effects, we focused on the use of salinomycin concentrations which only cause minor cytotoxicity. Finally, a syngenic mouse model for metastasis was utilized to prove the efficacy of salinomycin against tumor dissemination.

### Salinomycin treatment effectively hampers migration in cancer cells

**Figure 1:**
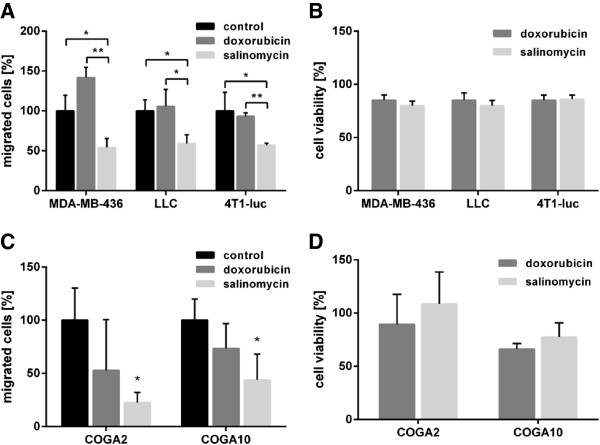
**Salinomycin treatment effectively hampers migration in cancer cells. A)** Boyden chamber migration assays of different cancer cell lines during treatment. MDA-MB-436, LLC and 4T1-luc cells were treated for 18 h with 10 μM, 0.5 μM and 0.5 μM doxorubicin or 0.05 μM, 0.5 μM and 0.05 μM salinomycin, respectively. The number of migrated mock-treated (control) cells was set to 100%. (Student’s t-test, two-tailed, *p < 0.05; **p < 0.01). **B)** Cell viability upon doxorubicin or salinomycin treatment. MDA-MB-436, LLC and 4T1-luc cells were treated for 18 h with afore mentioned concentrations of doxorubicin or salinomycin. Cell viability was determined by a CellTiter Glo assay and normalized to mock-treated control cells. **C)** Boyden chamber migration assays of primary colon cancer cells. COGA2 and COGA10 cells were treated for 18 h with either doxorubicin (10 μM and 10 μM, respectively) or salinomycin (2.5 μM and 5 μM, respectively). The number of migrated mock-treated (control) cells was set to 100%. (Student’s t-test, two-tailed, salinomycin compared to control; *p < 0.05). **D)** Cell viability upon doxorubicin or salinomycin treatment. COGA2 and COGA10 cells were treated for 18 h with afore mentioned concentrations of doxorubicin or salinomycin. Cell viability was determined by a CellTiter Glo assay and normalized to mock-treated control cells.

To examine whether salinomycin has a strong anti-migratory effect on cells derived from several different cancer types, we performed Boyden chamber assays in which we treated the human breast cancer cell line MDA-MB-436, the murine lung cancer cell line Lewis lung carcinoma (LLC) and the murine breast cancer cell line 4T1-luc (i.e. 4T1 cells stably expressing firefly luciferase) with low concentrations of doxorubicin or salinomycin. All materials and methods are described in detail in Additional file 1: Supplementary materials and methods. Thereby, the concentrations of salinoymcin and doxorubicin were chosen to cause a low and comparable cytotoxicity (Figure 1B and Figure 1D). In all salinomycin-treated cells migration was significantly reduced as compared to mock- or doxorubicin-treated cells (Figure 1A), indicating that the inhibitory effect of salinomycin on migration is not due to unspecific cytotoxicity. Apart from the breast and lung cancer cell lines, we analyzed the migratory capability of the primary low passaged colon cancer cell lines COGA2 and COGA10, which were derived from colon cancer patients as previously described by Vecsey-Semjen *et al*. [9]. Interestingly, salinomycin treatment significantly inhibited the migration of these primary colon cancer cells as compared to mock treatment (Figure 1C).

### Time-lapse microscopy reveals an inhibition of the motility of MDA-MB-436 cells upon salinomycin treatment

**Figure 2:**
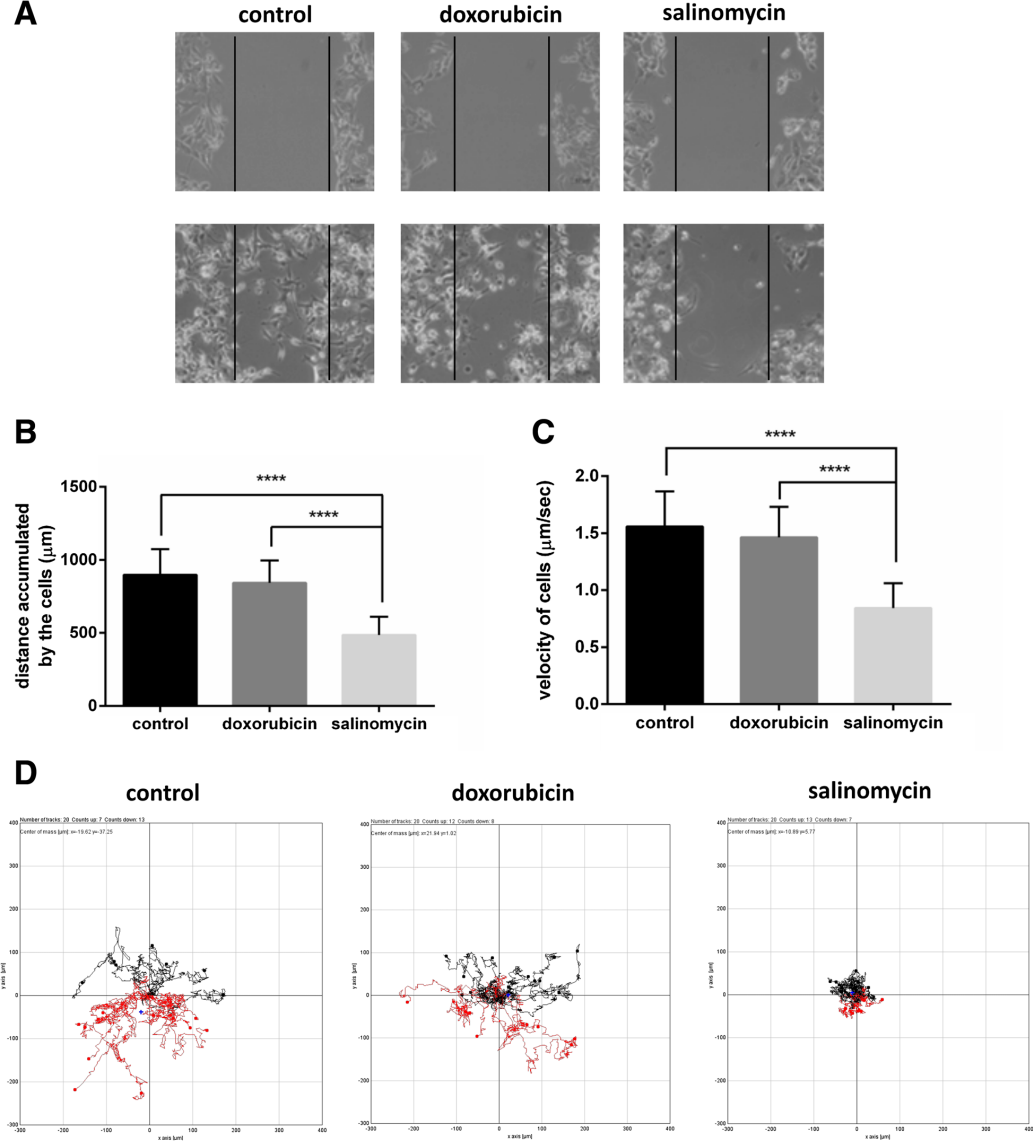
**Time-lapse microscopy reveals an inhibition of the motility of MDA-MB-436 cells upon salinomycin treatment. A)** Wound healing assay of MDA-MB-436 cells. Cells were treated either with mock (control), doxorubicin (0.05 μM) or salinomycin (0.05 μM) and monitored for 72 h. The same cells as for the wound healing assay were used for time-lapse microscopy. Manually tracked cells were analyzed for **B)** the accumulated distance, **C)** the velocity and **D)** the direction of movement. (Student’s t-test, two-tailed, ****p < 0.0001).

Moreover, we analyzed the effect of salinomycin on the cell motility of MDA-MB-436 cells in more detail using time-lapse microscopy. In a scratch assay, mock- (control) as well as doxorubicin-treated cells migrated into the scratch, whereas salinomycin treatment prevented wound closure (Figure 2A and Supplement videos^a^). Importantly, concentrations of salinomycin and doxorubicin were chosen to cause a comparable low cytotoxicity of less than 10% (Additional file 2: Figure S1). Subsequent analysis of individually tracked cells revealed that the accumulated distance (Figure 2B) and the velocity (Figure 2C) of salinomycin-treated cells were significantly reduced as compared to mock- or doxorubicin-treated cells. This effect became even more evident when looking at the moving direction of the cells treated with salinomycin (Figure 2D). In addition, we performed a scratch assay with the human breast cancer cell line MDA-MB-231 to further demonstrate the potent inhibitory effect of salinomycin on cancer cell motility (Additional file 3: Figure S2). Moreover, the salinomycin-induced changes on the cell morphology and cytoskeleton of MDA-MB-436 were analyzed by immunofluorescence microscopy (Additional file 4: Figure S3). These findings clearly demonstrate the strong anti-migratory effects of salinomycin on various cancer cells *in vitro*.

### Metastasis formation is reduced by salinomycin treatment in a syngenic intravenous mouse tumor model

**Figure 3:**
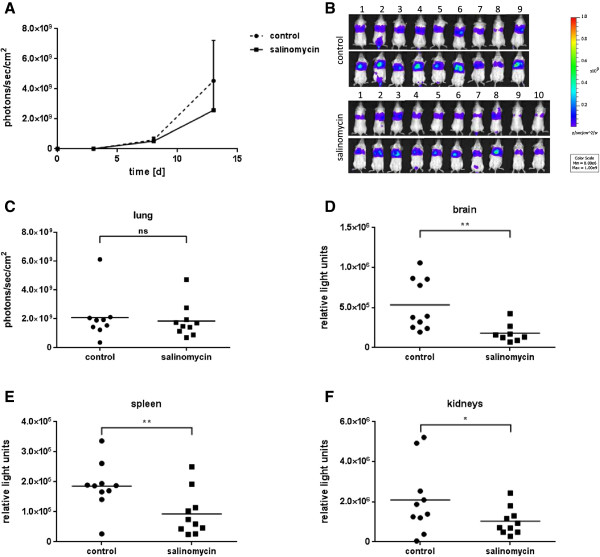
**Metastasis formation is reduced by salinomycin treatment in a syngenic intravenous mouse tumor model. A)** Tumor growth of 4T1-luc tumors in the lung. 1×10^5^ 4T1-luc cells were intravenously injected into 10 female BALB/c mice per group. Animals were treated either with mock (control) or 5 mg/kg salinomycin on day 0, 3, 6 and 9. Tumor growth was monitored for 13 days at indicated time points using bioluminescence imaging. **B)** Live imaging of 4T1-luc tumors in mouse lungs. Bioluminescence images were taken in dorsoventral (upper panel) and ventrodorsal (lower panel) position and presented for day 13. Signal intensities are displayed as photons/second/cm^2^ (scale bar). **C)***Ex vivo* imaging of 4T1-luc tumor-bearing lungs. Mice were euthanized on day 13 and the resected lungs of mock- (control) and salinomycin-treated mice were imaged. Signal intensities are displayed as photons/second/cm^2^. (Student’s t-test, two-tailed; ns = not significant). Metastases in 4T1-luc tumor-bearing mice. **D)** Brain, **E)** spleen and **F)** kidneys were analyzed for metastases using an *ex vivo* luciferase assay. (Student’s t-test, one-tailed; *p < 0.05; **p < 0.01).

Based on these observations, we raised the question whether salinomycin is able to prevent metastasis *in vivo*. Hence, we injected 4T1-luc cells into the tail vein of BALB/c mice and treated them with salinomycin at indicated time points. Primary tumor formation in the lungs was monitored *in vivo* using bioluminescence imaging as described previously [10]. Interestingly, there was no significant effect of salinomycin treatment on primary tumor formation and growth (Figure 3A). Bioluminescence images of the tumor-bearing mice (Figure 3B) and analysis of the respective resected 4T1-luc tumor-bearing lungs (Figure 3C) of mock- and salinomycin-treated mice further confirmed that there was no major effect on primary tumor growth in the lungs. However, when analyzing the metastatic tumor burden in other organs using an *ex vivo* luciferase assay metastases were significantly reduced upon salinomycin treatment in brain (Figure 3D), spleen (Figure 3E) and kidneys (Figure 3F).

In summary, salinomycin had considerable inhibitory effects on cell migration in several different cancer cell lines including MDA-MB-436 (breast), MDA-MB-231 (breast), 4T1-luc (breast), LLC (lung), COGA2 (colon) and COGA10 (colon) when applied at low dose. Selective targeting of CSC and induction of oxidative stress have been suggested to be responsible for this anti-migratory effect [4,6]. Verdoodt *et al*. have recently demonstrated that salinomycin induces autophagy in colon and breast cancer cells [11], which might also hinder cells to migrate as autophagy has been shown to inhibit migration in hepatitis B virus-associated hepatocellular carcinoma and in HeLa cells [12,13]. It is furthermore well established that salinomycin treatment leads indirectly to an influx of calcium ions into the cytoplasm most likely *via* the potassium/calcium antiporter [14,15]. Gradients of calcium are crucial for polarized cell migration in mesenchymal cells. In the leading edge protrusion transient calcium flickers are induced by chemokines and regulate directional decision making [16]. Similarly, it was speculated that detachment of the trailing edge is also calcium dependent [17]. Recently, Witze *et al*. [18] demonstrated that Wnt5a induces ER mobilization to the trailing edge in migrating cells controlling calcium signaling *via* ER tubules, finally resulting in the activation of calpain proteases and substrate detachment. The collapse of the fine tuned calcium balance at both cellular edges by additional influx of calcium induced by salinomycin might lead to a breakdown of the calcium gradients hindering cells to execute directed movements.

In some of the previous studies in which they treated cells with salinomycin for migration experiments [4,7,8], relatively high salinomycin concentrations were used so that in our view the inhibitory effect on cell motility cannot be exclusively attributed to anti-migratory effects of salinomycin but rather to unspecific cytotoxicity. Here, we compared salinomycin- with doxorubicin-treated cells at concentrations resulting in approximately 85 – 90% viable cells. Since salinomycin treatment significantly reduced the migratory capacity of the breast and lung cancer cell lines to 50 – 55% in contrast to doxorubicin treatment, we conclude that salinomycin is able to inhibit cancer cell migration at low-toxic doses, independent of the induced cytotoxicity. In case of the migratory potential of the primary colon cancer cell lines, salinomycin treatment was not significantly superior to doxorubicin treatment due to higher standard deviations. However, in COGA2 cells treatment with non-toxic concentrations of salinomycin (cell viability of approximately 100%) reduced the number of migrated cells to 20 – 25%. The salinomycin concentration used for COGA10 cells (cell viability of approximately 75%) could at least reduce the number of migrated cells to 40 – 45%, albeit cytotoxic effects cannot be completely excluded in this setting. Exemplarily, we performed time-lapse microscopy of MDA-MB-436 cells to directly monitor the immediate effects of salinomycin on cell motility, i.e. the distance, the velocity and the direction of migrating cells, on a single-cell level. The videos taken from these experiments further underline the quantitative analyses of the cell motility. Hence, we showed, to our knowledge for the first time, that salinomycin inhibits migration of breast cancer cells and primary colon cancer cells independent of the induction of cell death. Consequently, we sought to explore the efficacy of salinomycin on tumor dissemination *in vivo*. Gupta *et al*. pre-treated 4T1 cells with salinomycin before they injected them intravenously into mice. After three weeks they obtained a smaller tumor burden upon salinomycin treatment in the lungs as determined by the lung tumor surface [1]. In this study, we analyzed for the first time metastasis formation of intravenously injected firefly luciferase expressing 4T1-luc cells which were not pre-treated with salinomycin. The rationale behind this was to monitor the primary tumor formation in the lungs *via* bioluminescence imaging as well as to detect metastases in other organs *via* an *ex vivo* luciferase assay. Of note, metastasis formation in brain, spleen and kidneys from primary 4T1-luc tumors in the lungs was considerably reduced by salinomycin treatment, even though the growth of the primary lesion was not significantly hampered. Thus, our *in vitro* and *in vivo* results clearly demonstrate that salinomycin - initially a cancer stem cell-specific drug – inhibits the migration of various cancer cells and prevents tumor dissemination in mice. These findings raise hope for improved treatment options for cancer patients in the future.

## Endnote

^a^Supplement videos of the wound healing assay of MDA-MB-436 cells treated either with mock (control), doxorubicin or salinomycin are available on our homepage: http://www.cup.lmu.de/pb/aks/ewagner/projects.html.

## Abbreviations

CSC: Cancer stem cells.

## Competing interests

The authors declare that they have no competing interests.

## Authors’ contributions

The study was conceived by AR. FK and AR designed the study and analyzed the data. FK, AdH, PO and AnH performed the experiments. FK and AR wrote the manuscript. EW provided conceptual advice. All authors read and approved the manuscript.

## Supplementary Material

Additional file 1 Supplementary materials and methods. Click here for file

Additional file 2: Figure S1 Cell viability upon doxorubicin or salinomycin treatment. MDA-MB-436 cells were treated for 72h with 0.05μM doxorubicin or 0.05μM salinomycin. Cell viability was determined by a CellTiter Glo assay and normalized to mock-treated control cells. Click here for file

Additional file 3: Figure S2 Wound healing assay of MDA-MB-231 cells. Cells were treated either with mock (control), doxorubicin (0.05μM) or salinomycin (0.10μM) and monitored for 48h using the IncuCyte ZOOM 40008 instrument (ESSEN BioScience) (upper panel). The relative wound density was analyzed using the IncuCyte ZOOM 2013A software (lower panel). (Values are stated as mean ± SEM, student’s t-test, two-tailed, ****p < 0.0001). Click here for file

Additional file 4: Figure S3 Immunofluorescence microscopy of salinomycin-treated MDA-MB-436 cells. Cells were grown to a confluency of 80 – 90% and a scratch was placed. Subsequently, cells were treated with 500nM salinomycin for 24h and stained with anti-vinculin antibody (green), rhodamine phalloidin (F-actin, red) and DAPI (nuclei, blue). Representative pictures are shown. Click here for file
