# Supplementary material for: Salinomycin treatment reduces metastatic tumor burden by hampering cancer cell migration
Source: Mol Cancer. 2014 Jan 27;13:16. doi: 10.1186/1476-4598-13-16 (PMC3909296; doi:10.1186/1476-4598-13-16)
Supplement: Additional file 1 — Supplementary materials and methods. [file 1476-4598-13-16-S1.pdf]

Supplementary materials and methods

Reagents

Salinomycin (S6201) and doxorubicin hydrochloride (D1515) were obtained from Sigma-Aldrich. For immunofluorescence staining of vinculin and F-actin the following reagents were used: mouse anti-vinculin antibody (Clone hVIN-1) (V9264, Sigma-Aldrich) and Rhodamine Phalloidin (R415, Life Technologies).

Cell culture

The human breast cancer cell lines MDA-MB-436 and MDA-MB-231, the murine breast cancer cell line 4T1-luc (4T1 cells stably expressing firefly luciferase) and murine Lewis lung carcinoma cells (LLC) were cultivated according to supplier's instructions (ATCC). The primary low passaged colon cancer cell lines COGA2 and COGA10 were characterized by Vecsey-Semjen *et al*. and cultured as previously described [1].

Cell viability assay

For cytotoxicity experiments cells were seeded on 96-well plates at a density of 5000 cells per well. After 24h cells were incubated with respective drugs. Subsequently, a CellTiter Glo (Promega) assay was performed according to manufacturer's protocol.

Cell viability was normalized to the respective mock-treated control cells and presented as percent of control. All experiments were done in triplicates.

Boyden chamber migration assay

Transwells (8µm pore size, Millipore) were placed in 24-well plates containing 10% FCS in medium plus or minus doxorubicin or salinomycin. Cells were suspended in 250µl serum free medium plus or minus chemotherapeutic drugs, added to the top of each chamber and incubated for 18h. Subsequently, chambers were washed and cells were removed from the upper side of the chamber with a cotton swab. Migrated cells were fixed and stained using the cell stain solution (Chemicon International). The average number of migrated cells from 15 representative fields (three replicates per condition) was counted under a phase contrast microscope. For treatment experiments only drug concentrations causing approximately 10 - 15% cell death (as determined by a cell viability assay) were used.

Time-lapse microscopy and wound healing assay

Time-lapse microscopy was performed utilizing a live-cell imaging setup consisting of a microscope (Carl Zeiss MicroImaging) equipped with a Pln Apo 10x/0.45 DICII objective, motorized scanning table and a stage incubator at 37°C without CO_2. Images were captured with an AxioCamMR Rev3 camera using the Axiovision Rel 4.8 software for microscope control and data acquisition. For wound healing assays MDA-MB-436 cells were seeded in 24-well plates and grown to 90 – 95% confluency.

Subsequently, a scratch was placed in the middle of the well with a sterile 200µl pipette tip (Eppendorf). After washing once with serum-free media, the respective treatments were performed. Media with FCS was added to control wells and media with different drugs was added to respective wells before starting the time-lapse imaging. The images were captured every 15min for 72h. Each reading was performed in triplicates. 10 random cells/ well, i.e. 30 random cells per condition, were manually tracked using ImageJ [2] and the data was analyzed using the 'chemotaxis and migration tool' plugin for ImageJ.

Wound healing assay of MDA-MB-231 cells

MDA-MB-231 cells were seeded in 96-well ImageLock plates (ESSEN BioScience) and grown to 90 – 95% confluency. Subsequently, a scratch was placed in the middle of the wells with the WoundMaker (ESSEN BioScience). After washing with phosphate buffered saline, the respective treatments were performed. Cells were monitored for 48h using the IncuCyte ZOOM 40008 instrument and analyzed with the IncuCyte Zoom 2013A software (ESSEN BioScience).

Immunofluorescence microscopy

Cells were seeded in 8-well chamber slides. At a confluency of 80 – 90% a scratch was placed in each well, cells were treated with 500nM salinomycin for 24h and fixed with 4% paraformaldehyde. Subsequently, cells were blocked and permeabilized with 10% FCS and 0.1% Triton X-100. Cells were then incubated with the indicated primary antibodies

overnight. DAPI was used for counterstaining the nuclei and images were captured using the 20x objective of the Axiovert 200 microscope (Carl Zeiss) and analyzed using AxioVision software (Carl Zeiss). Representative images are shown.

Animal experiments

All animal experiments were approved by the local ethical committee and performed according to the guidelines of the German law of protection of animal life.

Intravenous 4T1-luc mouse model

$1x10^5$ 4T1-luc cells were injected intravenously into the tail vein of 20 female BALB/c mice. 10 animals were treated either with mock (DMSO in phosphate buffered saline) or 5mg/kg salinomycin (2mg/ml in DMSO stock solution was diluted in phosphate buffered saline) on day 0, 3, 6 and 9. Tumor growth was monitored for 13 days at indicated time points using bioluminescence imaging (BLI). BLI was performed as described previously [3]. One mouse in the control group was missing for the last BLI (day 13) as this mouse had to be sacrificed earlier due to severe medical condition. At the end of the experiment mice were sacrificed and the mouse organs were resected for subsequent experiments. The lungs were weighed and the other indicated organs were subjected to an *ex vivo* luciferase assay in order to determine the metastatic tumor burden. For this purpose the obtained tissue was pulverized in liquid nitrogen and an aliquot homogenized in cell lysis buffer (Promega) using a

tissue and cell homogenizer (FastPrep-24, MP Biomedicals). Luciferase activity was quantified as described previously [4].

Statistical analysis

All values are stated as mean ± SD unless otherwise indicated. For statistical analysis student's t-tests were performed (* $p < 0.5$; ** $p < 0.01$; *** $p < 0.001$; **** $p < 0.0001$). Outliers were identified using the ROUT (robust regression and outlier removal) method of GraphPad Prism.

References

1. Vecsey-Semjen B, Becker KF, Sinski A, Blennow E, Vietor I, Zatloukal K, Beug H, Wagner E, Huber LA: **Novel colon cancer cell lines leading to better understanding of the diversity of respective primary cancers.** *Oncogene* 2002, **21:**4646-4662.
2. **ImageJ, U.S. National Institutes of Health, Bethesda, Maryland, USA** [http://rsb.info.nih.gov/ij/]
3. Wiedmann RM, von Schwarzenberg K, Palamidessi A, Schreiner L, Kubisch R, Liebl J, Schempp C, Trauner D, Vereb G, Zahler S, et al: **The V-ATPase-inhibitor archazolid abrogates tumor metastasis via inhibition of endocytic activation of the Rho-GTPase Rac1.** *Cancer Res* 2012, **72:**5976-5987.
4. Schaffert D, Troiber C, Salcher EE, Frohlich T, Martin I, Badgujar N, Dohmen C, Edinger D, Klager R, Maiwald G, et al: **Solid-phase synthesis of sequence-defined T-, i-, and U-shape polymers for pDNA and siRNA delivery.** *Angew Chem Int Ed Engl* 2011, **50:**8986-8989.
