# Supplementary material for: Salinomycin treatment reduces metastatic tumor burden by hampering cancer cell migration
Source: Mol Cancer. 2014 Jan 27;13:16. doi: 10.1186/1476-4598-13-16 (PMC3909296; doi:10.1186/1476-4598-13-16)
Supplement: Additional file 2: Figure S1 — Cell viability upon doxorubicin or salinomycin treatment. MDA-MB-436 cells were treated for 72h with 0.05μM doxorubicin or 0.05μM salinomycin. Cell viability was determined by a CellTiter Glo assay and normalized to mock-treated control cells. [file 1476-4598-13-16-S2.pdf]

Supplement Figure S1)

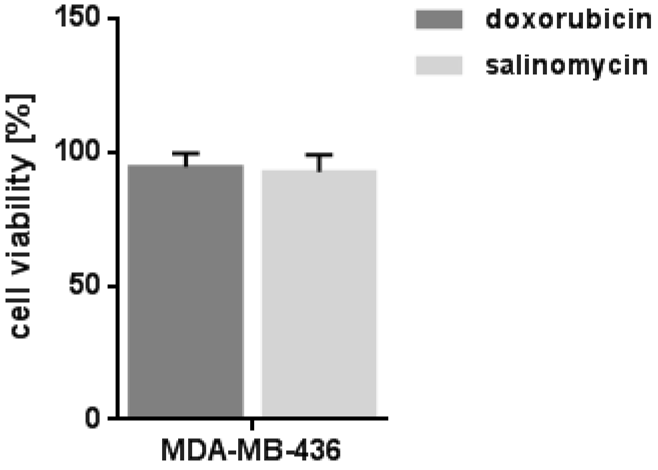

Cell viability upon doxorubicin or salinomycin treatment. MDA-MB-436 cells were treated for 72h with 0.05µM doxorubicin or 0.05µM salinomycin. Cell viability was determined by a CellTiter Glo assay and normalized to mock-treated control cells.
