# Supplementary material for: Salinomycin treatment reduces metastatic tumor burden by hampering cancer cell migration
Source: Mol Cancer. 2014 Jan 27;13:16. doi: 10.1186/1476-4598-13-16 (PMC3909296; doi:10.1186/1476-4598-13-16)
Supplement: Additional file 3: Figure S2 — Wound healing assay of MDA-MB-231 cells. Cells were treated either with mock (control), doxorubicin (0.05μM) or salinomycin (0.10μM) and monitored for 48h using the IncuCyte ZOOM 40008 instrument (ESSEN BioScience) (upper panel). The relative wound density was analyzed using the IncuCyte ZOOM 2013A software (lower panel). (Values are stated as mean ± SEM, student’s t-test, two-tailed, ****p < 0.0001). [file 1476-4598-13-16-S3.pdf]

Supplement Figure S2)

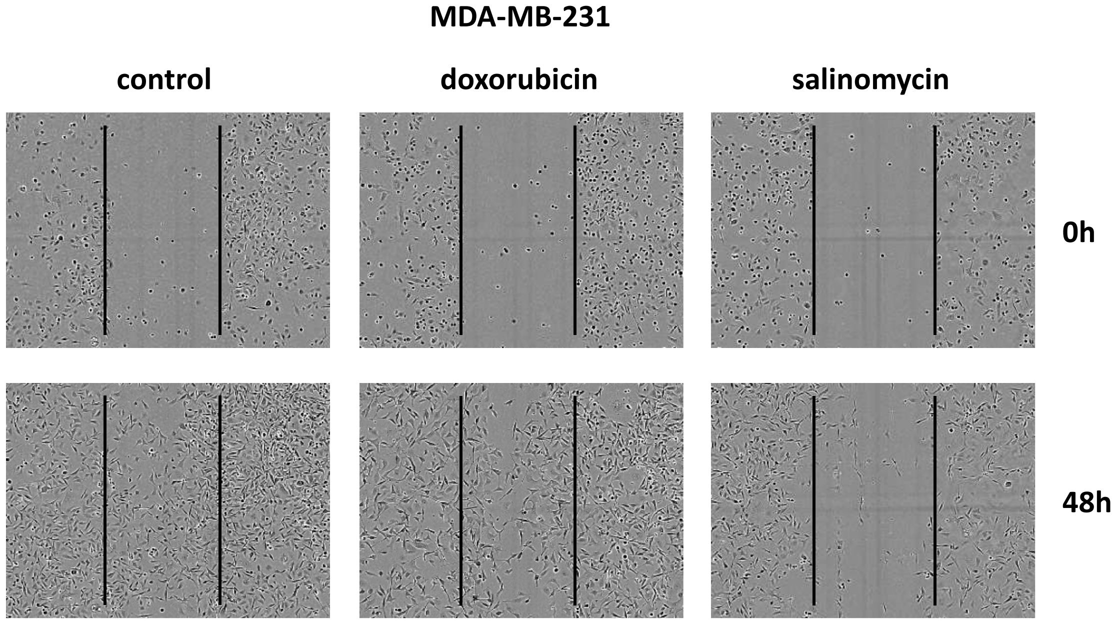

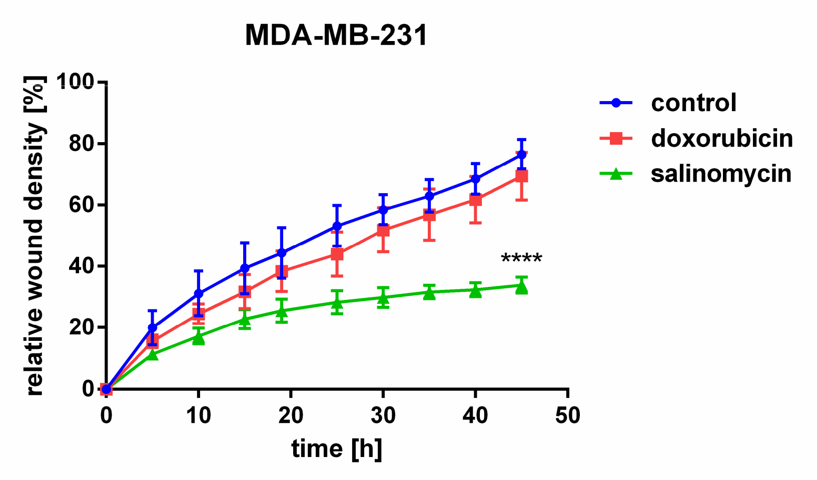

Wound healing assay of MDA-MB-231 cells. Cells were treated either with mock (control), doxorubicin (0.05µM) or salinomycin (0.10µM) and monitored for 48h using the IncuCyte ZOOM 40008 instrument (ESSEN BioScience) (upper panel). The relative wound density was analyzed using the IncuCyte ZOOM 2013A software (lower panel). (Values are stated as mean ± SEM, student's t-test, two-tailed, ****$p < 0.0001$)
