# Supplementary material for: Salinomycin treatment reduces metastatic tumor burden by hampering cancer cell migration
Source: Mol Cancer. 2014 Jan 27;13:16. doi: 10.1186/1476-4598-13-16 (PMC3909296; doi:10.1186/1476-4598-13-16)
Supplement: Additional file 4: Figure S3 — Immunofluorescence microscopy of salinomycin-treated MDA-MB-436 cells. Cells were grown to a confluency of 80 – 90% and a scratch was placed. Subsequently, cells were treated with 500nM salinomycin for 24h and stained with anti-vinculin antibody (green), rhodamine phalloidin (F-actin, red) and DAPI (nuclei, blue). Representative pictures are shown. [file 1476-4598-13-16-S4.pdf]

Supplement Figure S3)

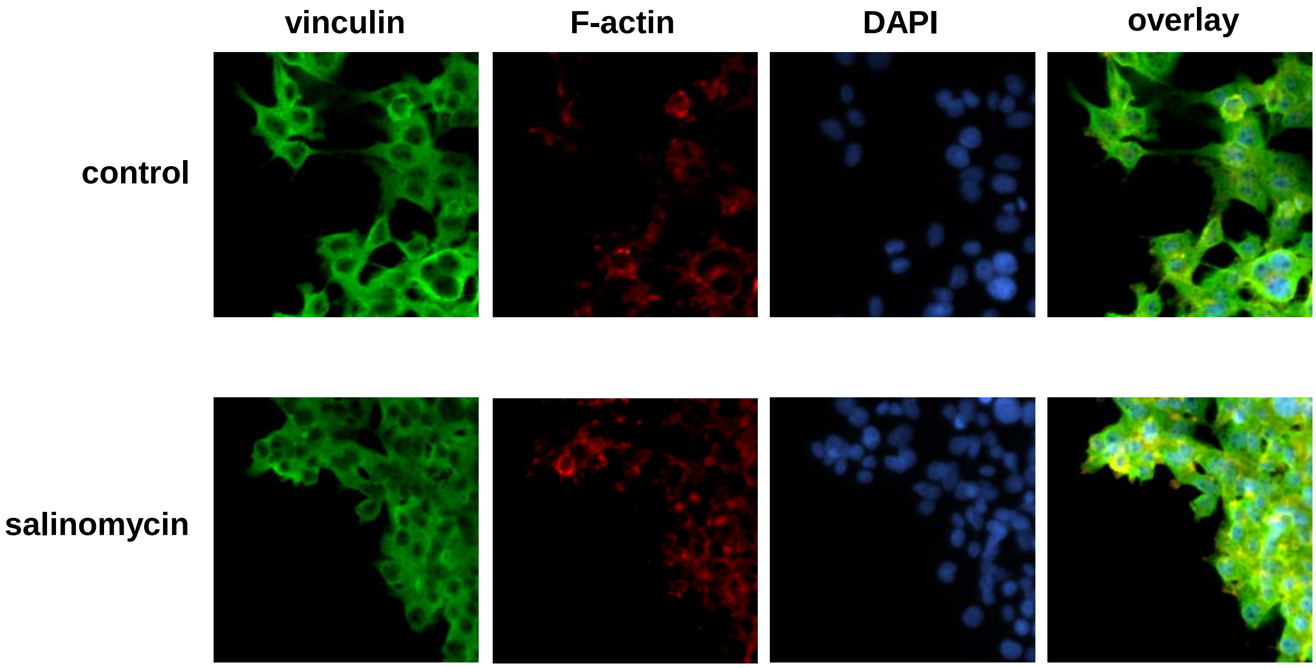

Immunofluorescence microscopy of salinomycin-treated MDA-MB-436 cells. Cells were grown to a confluency of 80 – 90% and a scratch was placed. Subsequently, cells were treated with 500nM salinomycin for 24h and stained with anti-vinculin antibody (green), rhodamine phalloidin (F-actin, red) and DAPI (nuclei, blue). Representative pictures are shown.
